# Comparison of Laparoscopic Varicocelectomy and Microsurgical Varicocelectomy with Internal Spermatic Vein-Superficial Epigastric Vein Bypass in Adolescent Patients

**DOI:** 10.3390/children13010077

**Published:** 2026-01-03

**Authors:** Dino Papeš, Zenon Pogorelić

**Affiliations:** 1 Department of Pediatric Surgery, University Hospital Centre Zagreb, 10000 Zagreb, Croatia; 2 Department of Surgery, School of Medicine, University of Zagreb, 10000 Zagreb, Croatia; 3 Department of Pediatric Surgery, University Hospital of Split, 21000 Split, Croatia; 4 Department of Surgery, School of Medicine, University of Split, 21000 Split, Croatia

**Keywords:** varicocele, adolescents, laparoscopic varicocelectomy, microsurgical varicocelectomy, venous bypass, testicular hypotrophy, semen parameters

## Abstract

Background/Objectives: Varicocele is a common cause of testicular hypotrophy and impaired semen quality in adolescents. Laparoscopic varicocelectomy (LV) and microsurgical varicocelectomy (MV) with internal spermatic vein-superficial epigastric vein bypass are established treatment options. This study aimed to compare clinical outcomes, complication rates, and functional recovery between LV and MV in adolescents. Methods: A retrospective two-center analysis was conducted on adolescents who underwent LV or MV between 2019 and 2024. Primary outcomes included postoperative complications, recurrence, testicular volume recovery, and semen parameter improvement. Secondary outcomes included operative time, hospital stay, and return to full activity. Statistical significance was set at *p* < 0.05. Results: A total of 430 patients met the inclusion criteria (270 LV, 160 MV). LV had a significantly shorter operative time (15 ± 5.1 min vs. 55.5 ± 6.4 min; *p* < 0.0001). There were no significant differences in hospital stay (*p* = 0.28), postoperative hematoma (*p* = 0.06), hydrocele (*p* = 0.06), or recurrence rates (*p* = 0.20). Full recovery of testicular volume occurred in 75.0% after LV vs. 70.6% after MV (*p* = 0.40). Overall semen improvement was 89.5% in LV vs. 100% in MV (*p* = 0.07). Normalization of oligospermia was significantly higher in the MV group (92.8% vs. 65.3%; *p* = 0.0048). Conclusions: Both LV and MV are safe and effective techniques for adolescent varicocele repair, with comparable complication and recurrence rates. LV offers significantly shorter operative time, whereas MV provides a superior improvement in semen parameters, suggesting a potential advantage of microsurgical repair in adolescents presenting with abnormal semen analysis.

## 1. Introduction

Varicocele is linked to impaired semen quality, reduced testicular growth, orchialgia, and hypogonadism, affecting around 15% of the general male population, 35% of men with primary infertility, and up to 80% of those with secondary infertility [1,2,3,4].

In the pediatric population, varicocele is reported in 8% of boys aged 11–14 years, with prevalence increasing to 14% among those aged 15–19 years. This age-related increase reflects the progressive nature of the condition, with varicocele-associated testicular hypotrophy being more commonly observed in older adolescents [5].

An association between varicocele and male subfertility was first reported more than 50 years ago [6], and along with hyperglycemia and smoking, it is the strongest independent predictor of male infertility [7].

Approximately 20% of adolescents diagnosed with varicocele are at risk of developing fertility problems, with the negative effects usually worsening over time [8,9]. Subfertility and infertility in varicocele are believed to result from cellular damage and sperm DNA fragmentation, both caused by increased scrotal temperatures and oxidative stress due to impaired clearance and the subsequent accumulation of reactive oxygen species [10,11].

The most widely accepted indications for surgical intervention in adolescents include reduced testicular volume (a discrepancy of more than 2 mL or 20% compared with the contralateral testis), symptomatic varicocele, and abnormal semen parameters in older adolescents [3,4]. However, these indications may expand to include molecular markers of sperm damage, such as the DNA fragmentation index (DFI), which measures sperm DNA integrity. Elevated DFI is linked to subfertility, an increased risk of miscarriage, and a higher incidence of congenital anomalies [1,12,13].

Surgical correction remains the standard of care for varicocele, with the two primary contemporary approaches being laparoscopic varicocelectomy (LV) and microsurgical varicocelectomy (MV) [2,3]. In addition to ligation during MV, a venous bypass may be created between the internal spermatic vein and either the inferior epigastric vein or a subcutaneous vein draining into the saphenous system (e.g., the superficial epigastric vein) to enhance testicular venous drainage. This venous bypass technique, first described four decades ago, offers a theoretical advantage by facilitating testicular cooling and improving the elimination of reactive oxygen species [14,15].

This study aims to compare outcomes after laparoscopic and microsurgical varicocelectomy with internal spermatic-to-superficial epigastric vein bypass in pediatric and adolescent patients.

## 2. Methods

### 2.1. Patients

**Figure 1:**
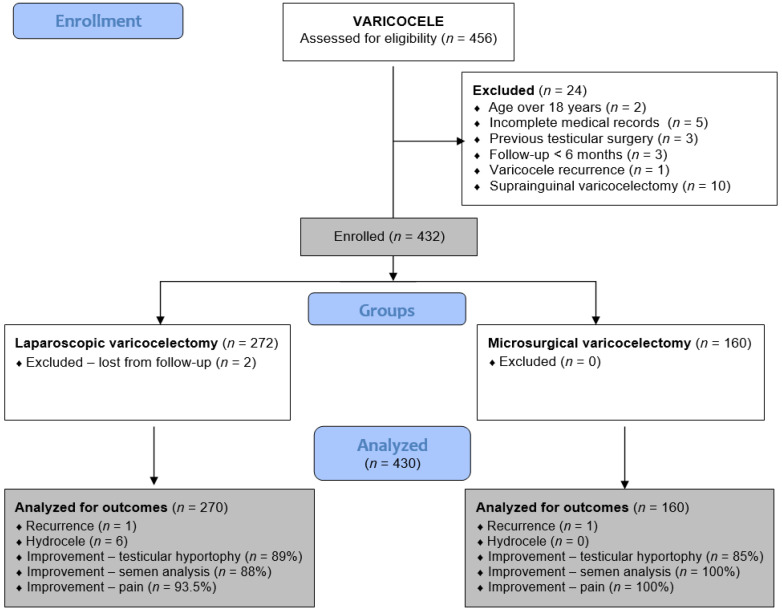
Flowchart of the study.

This retrospective analysis includes all adolescent patients who underwent laparoscopic or microsurgical varicocelectomy from 1 January 2019 to 31 December 2024 at two pediatric surgical centers. Patients at the University Hospital of Split received LV, while those at the University Hospital Centre Zagreb underwent MV. Adolescents who underwent LV or MV for symptomatic varicocele at either institution were included in the study. Exclusion criteria included open suprainguinal Palomo or Ivanissevich varicocelectomy, age over 18 years, previous testicular surgery, varicocele recurrence, follow-up less than six months, hyperglycemia, active smoking, or incomplete medical records. A flowchart of the study is shown in Figure 1.

### 2.2. Ethical Aspects

This study adhered to the Declaration of Helsinki of the World Medical Association and its subsequent amendments, as well as to comparable ethical standards. The Institutional Review Board of the University Hospital of Split approved the study (approval number: 500-03/23-01/227; date of approval: 27 November 2023). The Institutional Review Board of the University Hospital of Zagreb approved the study (approval number: 02/013 AG; date of approval: 4 April 2022).

### 2.3. Outcomes of the Study

The primary outcome measures were the incidence of complications (hydrocele, testicular atrophy), recurrence, and analysis of treatment outcomes (testicular volume recovery, improvement in semen analysis) between the LV and MV groups. Secondary outcome measures were the duration of the operation, time to discharge, and time to return to full activity.

### 2.4. Study Design

Data on patient demographics (age, height, weight), preoperative findings (varicoele grade, testicular volume, semen analysis when applicable, see below), operative procedure (surgery duration), intraoperative complications, and postoperative course (complications, recurrence rate, length of hospital stay, time to return to full activity) were collected and analyzed.

The indication for surgery was set based on preoperative clinical examination, testicular Doppler exam with testicular volume measurement, and semen analysis (when indicated, see below). The indications for varicocele surgery were symptomatic varicocele, testicular hypotrophy (loss of more than 20% of affected testicular volume compared with the contralateral testicle), abnormal semen analysis (in adolescents who had reached Tanner stage 5 of pubertal development), and bilateral varicocele. These indications were based on the European Urological Association guidelines [3].

Testicular volume was assessed clinically and quantified by ultrasonographic volumetry. The volume (in mL) was calculated by multiplying the maximal length of the testicle in the x, y, and z planes (in cm) by the corrective factor of 0.71 for an ellipsoid inscribed in a cuboid. Patients with a 20% difference in testicular volume underwent repeat measurement in 6 months, and surgery was indicated if the difference persisted. If the initial ultrasound volumetry revealed a volume difference of 50% or more, surgery was suggested without a control measurement.

Semen analysis was performed only in older adolescents aged 16–17 years, who reached Tanner V stage of sexual development. In accordance with institutional pediatric practice, it was not routinely performed in younger patients. Semen samples were collected after three days of abstinence and brought to the laboratory within 60 min of ejaculation. Patients were instructed to avoid cooling or heating the samples and to avoid semen analysis if ill or if nocturnal emission had occurred during abstinence. Semen analyses at both centers were performed according to routine clinical laboratory protocols based on World Health Organization (WHO) recommendations current at the time of analysis. Given the retrospective and multicenter design, formal inter-laboratory calibration was not performed, which may have introduced variability in semen parameter assessment. Overall improvement in semen analysis was defined as an increase in at least one abnormal semen parameter compared to baseline. Full normalization was defined as all measured parameters reaching reference values according to current WHO criteria. The assessed parameters included sperm concentration, progressive motility, and morphology.

### 2.5. Surgery

At each center, varicocele surgeries were performed by experienced pediatric surgeons according to standardized institutional protocols. Microsurgical procedures were performed by surgeons with formal microsurgical training. More than one surgeon was involved at each center. According to institutional pediatric surgical protocols, routine preoperative antibiotic prophylaxis was not administered, as the procedure was considered a clean surgery. In addition, no intraoperative or postoperative systemic anticoagulation or antiplatelet medications are administered.

#### 2.5.1. Laparoscopic Varicocelectomy

**Figure 2:**
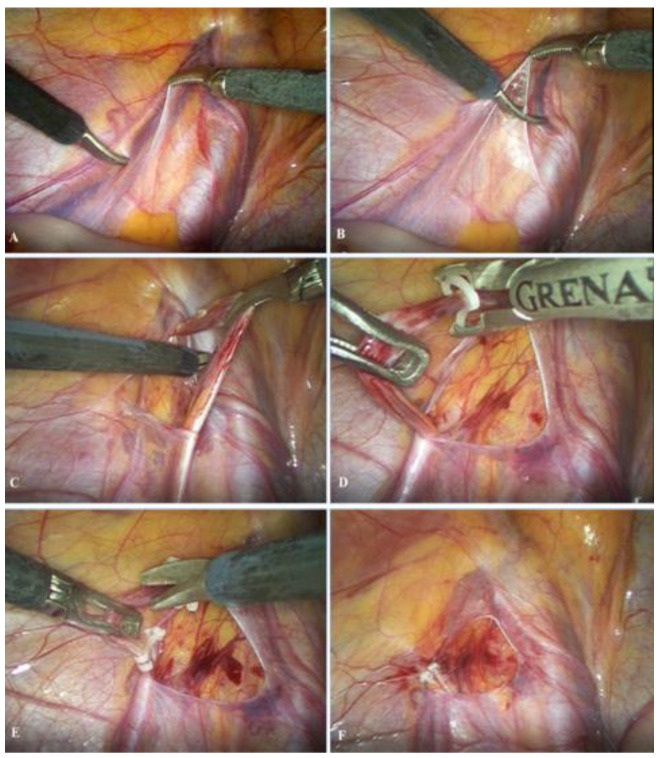
Laparoscopic varicocelectomy: (**A**) identification of spermatic vessels and vas deferens; (**B**) opening of the peritoneum; (**C**) mobilization and preparation of spermatic vessels; (**D**) placement of the polymer ligating clips; (**E**) resection of the spermatic vessels; (**F**) final outcome after resection of the spermatic vessels.

The patient is admitted on the day of the operation, which is performed under general anesthesia. A 5 mm supraumbilical incision is created, followed by insertion of a Veress needle to establish pneumoperitoneum at a pressure of 10–12 mmHg. A 5 mm trocar is then introduced through the supraumbilical incision, and a laparoscopic camera is inserted. Two additional 5 mm trocars are placed 1–2 cm inferior to the umbilicus along the left and right midclavicular lines, adjacent to the lateral border of the rectus abdominis muscle. After identification of the vas deferens and spermatic vessels (Figure 2A), the peritoneum is incised using laparoscopic scissors approximately 1 cm above the internal inguinal ring (Figure 2B). The spermatic vessels are subsequently isolated and carefully separated from the lymphatic vessels (Figure 2C), ligated with two polymer clips (Figure 2D) [16], and divided between the clips using endoscopic scissors (Figure 2E); alternatively, an ultrasonic scalpel may be used for vessel division [17]. Upon completion of the procedure, intra-abdominal pressure is reduced to 5–6 mmHg to inspect the operative field and confirm adequate hemostasis (Figure 2F). Finally, the trocars are removed, and the skin is closed with simple sutures.

#### 2.5.2. Microsurgical Varicocelectomy

The patient is admitted on the day of surgery. The surgery is performed under local, regional, or general anesthesia, according to the patient’s preference.

**Figure 3:**
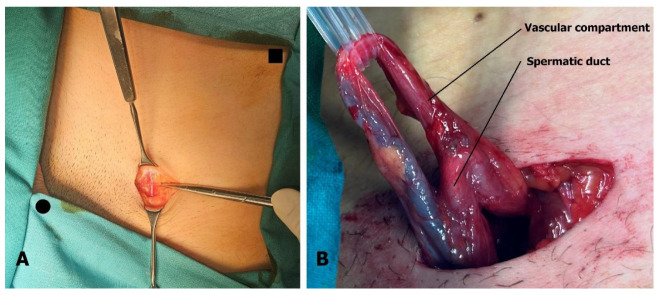
Microsurgical varicocelectomy: (**A**) Incision is situated above the external inguinal ring. The superficial epigastric vein is found in the subcutaneous tissue, usually below Scarpa’s fascia. Pubic tubercle (black dot) and superior anterior iliac spine (black square) are marked. (**B**) The spermatic cord is separated into two compartments, one containing the artery, veins, lymphatics, and nerves, and the other containing the spermatic duct with its blood vessels.

The 3 cm incision is located above the external inguinal ring. The superficial epigastric vein is identified within the incision (Figure 3A) and mobilized cranially and caudally to create a free segment 3 cm long. The spermatic cord is elevated on a sling, and if necessary, the external inguinal ring can be incised to facilitate this step. After ligating the external spermatic veins within the cremaster muscle or below the cord, the vas deferens and its blood vessels are dissected free, and the sling is repositioned to exclude the vas (Figure 3B).

**Figure 4:**
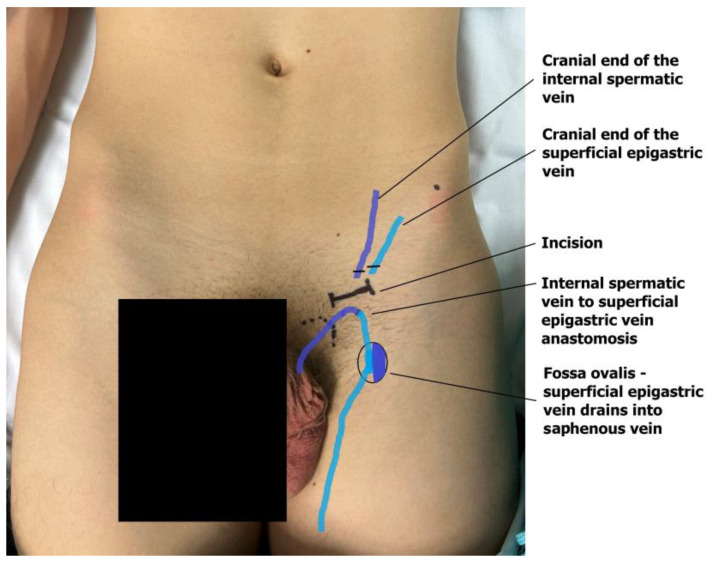
The anastomosis of the testicular end of the transected internal testicular vein and the caudal end of the superficial epigastric vein drains the testicular blood into the great saphenous vein bulb.

The dilated main internal spermatic veins (testicular veins) are dissected, and one is selected for the bypass. All internal spermatic veins are ligated and transected, and the selected vein is freed and transected 1–2 cm more cranially toward the internal inguinal ring to obtain a mobile segment. The artery and lymphatic ducts are dissected from the surrounding venous plexus and preserved. The nerves can also be transected if the indication for surgery was scrotal pain. The small veins around the artery are clipped or cauterized with bipolar diathermy. An end-to-end microvascular anastomosis is performed between the spermatic vein and the superficial epigastric vein using 9-0 or 10-0 nylon interrupted sutures, rerouting blood from the testicle to the saphenous vein (Figure 4). Following the patency test (refill test), the external ring is closed with one resorbable suture if needed [18,19]. The wound is then closed in layers, and the skin is sutured intradermally with fast-absorbing suture. Microsurgical varicocelectomy was performed via a subinguinal approach using a surgical microscope (Zeiss model S88) under ×6–×10 magnification.

### 2.6. Follow-Up

Both LV and MV are day-case surgeries, and patients are discharged on the same day or the following morning. Strolling is permitted on the first postoperative day, school attendance within 4–5 days, and full physical activity within 10–14 days. Minimal postoperative follow-up for inclusion in the study was 12 months. All patients underwent postoperative clinical and Doppler evaluation 3, 6, and 12 months after surgery. All patients who were operated on for abnormal semen analysis underwent control semen analysis three to six months after surgery.

### 2.7. Definitions

Full recovery of testicular volume was defined as a reduction in testicular volume asymmetry to less than 20% postoperatively, as measured on control Doppler examination. Partial recovery of testicular volume was defined as a reduction in asymmetry by at least 10% with a persisting volume difference of more than 20%.

Recurrence was defined as a clinically palpable varicocele grade III with reflux on Valsalva and reflux in duration of more than one second during Valsalva on Doppler examination while standing.

### 2.8. Statistical Analysis

Statistical analyses were conducted using MedCalc software (version 19.4; MedCalc Software Ltd., Ostend, Belgium). Data normality was assessed using the Kolmogorov–Smirnov test. Categorical variables are presented as frequencies and percentages, while continuous variables with a normal distribution are expressed as means ± standard deviations (SDs). Non-normally distributed continuous variables are reported as medians with interquartile ranges (IQRs). Comparisons of continuous variables were performed using the independent-sample *t*-test or the Mann–Whitney U test, as appropriate based on data distribution. Categorical variables were compared using the chi-square test, and Fisher’s exact test was applied when expected cell counts were low. Statistical significance was defined as *p* < 0.05.

## 3. Results

During the study period, 456 patients underwent surgery for varicocele: 284 underwent LV, and 172 underwent MV. After the exclusion criteria were applied, 270 and 160 patients remained for analysis in the LV and MV groups, respectively.

Demographic and preoperative data of the patients are shown in Table 1. The median age of all the patients was 16 years (range 11–18). The most common indication for surgery in both groups was testicular hypotrophy (*n* = 273; 63.5%), followed by an abnormal semen analysis (*n* = 123; 28.6%) and subjective discomfort or pain (*n* = 89; 20.7%), with 60 patients (13.9%) having multiple indications.

Median surgery duration (from incision to wound coverage) was significantly shorter for LV compared to MV (15 min vs. 55 min). All patients in LV were operated on under general anesthesia. In the MV group, the majority of patients chose general anesthesia. No anesthesia-related intraoperative or postoperative complications were observed in either group.

There were no intraoperative complications in the LV group. In the MV group, there were two intraoperative testicular artery injuries (1.3%). In one patient, the artery was ligated; the error was recognized immediately, and the artery was reconstructed by an end-to-end anastomosis. The anastomosis was patent, and postoperative Doppler showed symmetrical arterial flow in both testicles and no signs of atrophy. In another patient, the artery was injured tangentially and repaired with direct sutures. Normal distal refill was present. No statistically significant differences were found between the groups in terms of length of hospital stay, complications, and recurrence rate. In the MV group, two patients developed postoperative wounds and scrotal hematomas, which resolved spontaneously; none required intervention. Three of the 430 patients (0.7%) had recurrence (one in the LV and two in the MV group). Intraoperative and postoperative data of the patients included in the analysis are shown in Table 2.

Regarding discharge, all patients in both groups were discharged within 24 h of admission, and there were no re-admissions.

The rates of testicular hypotrophy recovery and symptom resolution were similar in both groups.

Overall semen analysis improvement was 89.5% (89/95 patients) in the LV group and 100% (28/28 patients) in the MV group (*p* = 0.07). In the MV group, improvement most commonly involved sperm concentration and progressive motility, while full normalization was achieved in 92.8% of patients with preoperative oligospermia. The percentage of patients with preoperative oligospermia who achieved normal postoperative semen analysis was significantly higher in the MV group (92%) than in the LV group (65%). Postoperative outcomes are shown in Table 3.

## 4. Discussion

In this retrospective multicenter study, laparoscopic and microsurgical varicocelectomy demonstrated comparable safety and effectiveness in adolescents undergoing surgery for symptoms or left testicular hypotrophy. Both techniques were associated with similar complication and recurrence rates, as well as comparable recovery of testicular volume, which was achieved in approximately 80% of patients. In a selected subgroup of older adolescents who underwent semen analysis, microsurgical repair was associated with a more pronounced improvement in semen parameters compared with laparoscopic repair. However, these findings should be interpreted with caution, given the baseline differences between groups, the limited sample size of the semen analysis subgroup, and the retrospective, center-based nature of the study.

Several operative techniques have been described for varicocele repair; however, laparoscopic varicocelectomy (LV) and microsurgical varicocelectomy (MV) are currently the most commonly applied approaches in adolescent surgical practice and were therefore the focus of the present study [2,3].

LV and MV are generally regarded as effective procedures, although recent analyses have suggested potential advantages of the microsurgical approach, including lower complication and recurrence rates and greater improvements in semen parameters [20,21,22,23,24,25,26,27]. Our findings are largely consistent with previously published comparative studies evaluating laparoscopic and microsurgical varicocelectomy, which have reported similar safety and recurrence profiles, with a tendency toward greater improvement in semen parameters following microsurgical repair [20,21,22,23]. These reported differences remain a matter of ongoing debate, particularly in adolescent populations.

In our analysis, postoperative complication rates did not differ significantly between LV and MV and remained within published ranges for microsurgical ligation (recurrence 1–5%, hydrocele 1–2%). Although MV can be performed under local or regional anesthesia, often cited as a practical advantage over LV, most adolescents in our cohort ultimately preferred general anesthesia regardless of the surgical technique.

As expected, operative time was significantly shorter for LV. Regarding clinical outcomes, both techniques achieved testicular volume recovery in approximately 75% of adolescents, consistent with previously reported rates of 60–80%. Postoperative semen parameters improved to a greater extent following MV, which aligns with findings from prior comparative studies.

Despite historically higher recurrence rates reported for LV compared with MV, this pattern was not observed in the present series. Recurrence after LV was markedly lower than typically reported (0.3% vs. 5–8%), while recurrence following MV was consistent with prior literature (1–2%) [3,23]. Previously described differences in recurrence rates may be influenced by anatomical variations in venous drainage and compression patterns described in classic angiographic studies [28,29,30,31]. Such factors may contribute to recurrence through collateral pathways that are not uniformly addressed by all surgical approaches [3,32].

This study identified significant variation in operative indications between the two participating centers, introducing potential selection bias. In one center, testicular hypotrophy was the predominant indication, whereas in the second center, hypotrophy and abnormal semen analysis were equally common, and multiple indications occurred more frequently. These differences reflect distinct referral patterns and diagnostic protocols. In particular, semen analysis was obtained selectively in Tanner stage V adolescents in one center, while it was routinely performed in all Tanner stage V patients in the other center, resulting in a higher proportion of patients with abnormal preoperative semen parameters and multiple indications for surgery. Such heterogeneity is inherent to multicenter retrospective studies and should be considered when interpreting comparative outcomes.

Based on the present analysis, MV and LV appear equally effective for adolescents undergoing varicocele repair for symptoms or left testicular hypotrophy, with comparable complication and recurrence rates. In the selected subgroup of adolescents with impaired semen parameters, MV was associated with a greater degree of postoperative improvement. The principal advantage of LV is its significantly shorter operative duration, whereas MV offers the option of regional or local anesthesia, although this was not a decisive factor for most patients in this cohort. Similar findings are reported in the literature.

Recent observational studies have demonstrated favorable seminal parameter improvements after laparoscopic varicocelectomy in patients with clinical varicocele, with significant increases in sperm count and motility following surgery [16,17,33]. Similarly, newer evidence on microsurgical varicocelectomy suggests significant enhancements in sperm capacitation and probability of generating pregnancy postoperatively, as well as substantial improvements in sperm count in infertile cohorts [34,35].

This study has several limitations. Its retrospective, non-randomized design introduces potential selection and information bias, particularly because each surgical technique was performed in a different center with differing referral patterns, institutional protocols, and preoperative indications. Consequently, the surgical approach was entirely center-specific and fully confounded by institutional practice patterns and diagnostic protocols. Under these conditions, multivariable adjustment or propensity score matching was not methodologically appropriate, as center and technique were perfectly collinear and could not be disentangled analytically. The two groups were not fully comparable at baseline, especially regarding varicocele grade and the frequency of multiple surgical indications. Semen analysis was available only for a selected subgroup of older adolescents, limiting the generalizability and direct comparability of fertility-related outcomes, particularly given the higher proportion of abnormal preoperative semen findings in the MV group. Follow-up duration, although adequate for detecting recurrence and early recovery, was insufficient to assess long-term fertility or paternity. Finally, differences in surgeon experience, institutional protocols, and inter-laboratory variability in semen analysis may have influenced the observed outcomes.

## 5. Conclusions

LV and MV appear comparably effective and safe for adolescents undergoing varicocele repair for symptoms or left testicular hypotrophy. Both techniques demonstrated similar complication and recurrence rates, and testicular volume recovery was achieved in an equivalent proportion of patients (80%). However, microsurgical repair yielded a more pronounced improvement in semen parameters, suggesting a potential advantage of microsurgical repair in adolescents presenting with abnormal semen analysis, although these findings should be interpreted in light of the limited sample size and retrospective study design and the inability to assess long-term fertility or paternity outcomes. The advantage of LV is its significantly shorter operation time, whereas MV can be performed under local or regional anesthesia.

## Figures and Tables

**Table 1:** Demographic and preoperative data of the patients included in the analysis (*n* = 430).

Variables	Laparoscopic Varicocelectomy (*n* = 270)	Microsurgical Varicocelectomy (*n* = 160)	*p*
Age (years)	15.8 ± 1.9	15.6 ± 1.8	0.280
Mean, SD, range	11–18	11–18	
BMI Mean, SD	21.7 ± 3.8	21.1 ± 3.1	0.090
Varicocele grade, *n* (%)			
Grade 3	135 (50.0)	126 (78.8)	<0.0001
Grade 2	123 (45.6)	34 (21.2)	<0.0001
Grade 1	12 (4.4)	0 (0)	0.007
Indication, *n* (%)			
Testicular hypotrophy	147 (54.4)	126 (78.8)	
- isolated	106	122	
- combined	41	4	<0.0001
Abnormal semen analysis	95 (35.2)	28 (17.5)	
- isolated	58	24	
- combined	37	4	0.0001
Symptoms	76 (28.1)	13 (8.1)	
- isolated	43	5	
- combined	33	8	<0.0001
Bilateral varicocele	4	1	0.655
Multiple indications	52 (19.3)	8 (5.0)	
Hypotrophy + oligospermia	19	0	<0.0001
Symptoms + oligospermia	11	4	
Hypotrophy + symptoms	15	4	
Hypotrophy + oligospermia + symptoms	7	0	

SD—standard deviation.

**Table 2:** Intraoperative and postoperative data of the patients included in the analysis (*n* = 430).

Variables	Laparoscopic Varicocelectomy (*n* = 270)	Microsurgical Varicocelectomy (*n* = 160)	*p*
Surgery duration (minutes)	15.0 ±5.1	55.5 ± 6.4	
mean ± SD, range	9–31	45–90	
Type of anesthesia; *n* (%)			
General	270 (100)	148 (92.5)	<0.0001
Spinal	0 (0)	10 (6.2)	<0.01
Local + ilioinguinal block	0 (0)	2 (1.3)	0.06
Time to discharge (days)	1 ± 0.01	1 ± 0.08	0.921
Mean ± SD, range	1–1	1–2	
Complications; *n* (%)			
Wound hematoma	0 (0)	2 (1.3)	0.06
Hydrocele	6 (2.2)	0 (0)	0.06
Recurrence; *n* (%)	1 (0.3)	2 (1.3)	0.2

SD—standard deviation.

**Table 3:** Postoperative outcomes after laparoscopic varicocelectomy (*n* = 430).

Variables	Laparoscopic Varicocelectomy (*n* = 270)	Microsurgical Varicocelectomy (*n* = 160)	*p*
Testicular hypotrophy	(*n* = 147)	(*n* = 126)	
Full recovery	114 (75.0)	89 (70.6)	0.401
Partial recovery	18 (13.8)	18 (14.3)	0.902
No recovery	15 (11.2)	19 (15.1)	0.301
Semen analysis	(*n* = 95)	(*n* = 28)	
Normal	64 (65.3)	26 (92.8)	0.004
Improved but below normal	21 (22.5)	2 (7.2)	0.070
No improvement	8 (12.2)	0 (0)	0.053
Symptoms	(*n* = 76)	(*n* = 13)	
No symptoms	63 (82.9)	12 (92.3)	0.411
Minor residual symptoms	8 (10.5)	1 (7.7)	0.705
No improvement	5 (6.6)	0 (0)	0.312

## Data Availability

The data assessed and reported here can be obtained from the authors upon reasonable request and following ethical and privacy principles.
